# Effect of food additives on key bacterial taxa and the mucosa-associated microbiota in Crohn’s disease. The ENIGMA study

**DOI:** 10.1080/19490976.2023.2172670

**Published:** 2023-02-27

**Authors:** J.J. Jimenez Loayza, S. Kang, L. Schooth, J. J. Teh, A. de Klerk, E. K. Noon, J. Zhang, J. Hu, A. L. Hamilton, A. Wilson-O’Brien, G. L. Trakman, W. Lin, J. Ching, L. Or, J.J.Y. Sung, J. Yu, S.C. Ng, M.A. Kamm, M. Morrison

**Affiliations:** a Frazer Institute, Faculty of Medicine, University of Queensland, Brisbane, Australia; b Department of Medicine and Therapeutics, the Chinese University of Hong Kong, Hong Kong, China; c State Key Laboratory of Digestive Diseases, Li Ka Shing Institute of Health Science, The Chinese University of Hong Kong, Hong Kong, China; d Microbiota I-Center (Magic), Hong Kong, China; e Department of Gastroenterology, St Vincent’s Hospital, Melbourne, Australia; f Department of Medicine, the University of Melbourne, Melbourne, Australia; g Lee Kong Chian School of Medicine, Nanyang Technological University, Singapore, Singapore; h Centre for Gut Microbiota Research, Faculty of Medicine, The Chinese University of Hong Kong, Hong Kong, China

**Keywords:** Gut microbiota, food additives, inflammatory bowel disease, *Proteus*, *Veillonella*, *Faecalibacterium prausnitzii*

## Abstract

Food additives have been linked to the pro-inflammatory microbial dysbiosis associated with Crohn’s disease (CD) but the underlying ecological dynamics are unknown. Here, we examine how selection of food additives affects the growth of multiple strains of a key beneficial bacterium (*Faecalibacterium prausnitzii*), axenic clinical isolates of proinflammatory bacteria from CD patients (*Proteus, Morganella*, and *Klebsiella* spp.), and the consortia of mucosa-associated microbiota recovered from multiple Crohn’s disease patients. Bacterial growth of the axenic isolates was evaluated using a habitat-simulating medium supplemented with either sodium sulfite, aluminum silicate, carrageenan, carboxymethylcellulose, polysorbate 80, saccharin, sucralose, or aspartame, intended to approximate concentrations found in food. The microbial consortia recovered from post-operative CD patient mucosal biopsy samples were challenged with either carboxymethylcellulose and/or polysorbate 80, and the bacterial communities compared to unchallenged consortia by 16S rRNA gene amplicon profiling. Growth of all *F. prausnitzii* strains was arrested when either sodium sulfite or polysorbate 80 was added to cultures at baseline or mid-exponential phase of growth, and the inhibitory effects on the Gram-negative bacteria by sodium sulfite were conditional on oxygen availability. The effects from polysorbate 80, saccharin, carrageenan, and/or carboxymethylcellulose on these bacteria were strain-specific. In addition to their direct effects on bacterial growth, polysorbate 80 and/or carboxymethylcellulose can drive profound changes in the CD mucosa-associated microbiota via niche expansion of *Proteus* and/or *Veillonellaceae* – both implicated in early Crohn’s disease recurrence. These studies on the interaction of food additives with the enteric microbiota provide a basis for dietary management in Crohn’s disease.

## Introduction

Inflammatory bowel diseases (IBD) are idiopathic, chronic conditions characterized by a dysregulated immune response resulting in inflammation and mucosal ulceration.^1^ Genetic susceptibility has long been considered necessary but not sufficient to trigger IBD onset and its recurrence, and now is widely accepted to involve alterations in the taxonomic and functional landscapes of the gut microbiota disrupting gut homeostasis.^2,3^ Extrinsic factors such as lifestyle, increased hygiene, drug exposure, diet, and intake of ultra-processed foods have coincided with the rising incidence and prevalence rates of IBD in Europe and North America over the last century.^1,4^ Furthermore, the Westernization of developing countries, where IBD was once rare, is now coincident with rapidly emerging and increasing incidence of IBD rates in South America, Africa, and Asia.^5,6^

The microbial dysbiosis considered a hallmark of IBD has long been recognized to be typified by reduced relative abundances of Gram-positive *Firmicutes-*affiliated lineages and increases in the relative abundance of Gram-negative *Proteobacteria-*affiliated lineages.^7,8^ Among the lineages of Gram positive bacteria depleted in IBD, *Faecalibacterium prausnitzii* is considered a key microbial biomarker,^9^ being one of the most abundant members of the gastrointestinal microbiota of healthy humans^10^ and thereby, a numerically predominant source of butyrate, which imparts positive pleiotropic effects on epithelial cell metabolism, immune system modulation, and cell repair. The bacterium has also been shown to produce other “anti-inflammatory” factors^11–13^ promoting epithelial cell integrity and immune tolerization. The expansion of the relative abundance of *Proteobacteria-*affiliated lineages in IBD has been associated with both host genetics^14^ and changes in nutritional ecology favoring the metabolic capabilities of at least some of these bacteria.^15^ In that context, we have used a novel *ex vivo* combination of microbe culture with metagenomic sequencing (MC-MGS) for use with tissue biopsy samples^16^ and recovered axenic isolates of *Proteobacteria*, including urease-positive isolates of *Proteus mirabilis, Morganella morganii*, and *Klebsiella pneumoniae* from post-operative CD subjects, which substantiates the identification of urea metabolism as a key guild within the dysbiotic microbiome associated with CD.^15^ Although longitudinal studies of IBD in the clinical setting are relatively scant, the presence of *F. prausnitzii* in post-operative CD patients is associated with remission at six months.^11,^^17^ We have also established that concomitant low *F. prausnitzii* abundance and detectable *Proteus* spp. is associated with a significantly heightened risk of disease recurrence.^18^ Indeed, Mondot et al.^19^ proposed that *Proteus* spp. – of the bacterial family *Morganellaceae*^20^ – be considered a biomarker for post-surgery CD recurrence and our recent studies have further validated that *Proteus mirabilis* can be a key bacterium driving CD inflammation.^21^ In that context, *Morganella morganii* can also be recovered from CD mucosa-associated microbiota^16,22^ and Machiels et al.^23^ have also recently reported that CD patients that suffer early post-operative recurrence possess elevated abundances of the *Negativicutes* and Fusobacteria on ileal mucosa. In summary, the gross imbalances in microbial taxa that typify CD dysbiosis are linked with genetic and/or ecological alterations that can manifest in disruptions to gut homeostasis, and key bacterial taxa affecting predisposition to CD remission or recurrence have been identified. However, the ecological drivers of these changes in microbial community dynamics are still largely unknown, and a key knowledge gap to improved CD treatment and management.

In that context, Levine et al.^24^ highlighted how diet and dietary components needed greater consideration as a trigger as well as a treatment of digestive diseases and disorders. The industrialization of food production and its diversification has influenced consumer choices, reflected in “Western diets” with reduced amounts of whole grains, plant-based non-starch polysaccharides, and other phytochemicals all known to promote gut microbial diversity. Processed foods also utilize various chemistries to minimize spoilage and extend shelf life, to enhance flavor, texture, and sensory properties of foods^25–27^ and/or as a substitute for other allergenic dietary components (e.g., using xanthan gum in place of gluten)^28^. These chemistries are holistically referred to as food additives and their impacts on the physicochemical and ecological characteristics of the stool microbiota has emerged as a new facet in the study of diet × microbiota interactions in the context of IBD onset and recurrence.^29–31^ However, and given that IBD is a “disease of tissue”, virtually nothing is known of how food additives might impact those bacterial taxa resident on mucosal tissue, and particularly for subjects with Crohn’s disease.

Here, we present our findings of the effects from common food additives on the growth of axenic isolates representing protective *(F. prausnitzii)* and clinical isolates of proinflammatory mucosa-associated bacteria *(Proteus mirabilis, Morganella morganii, Escherichia fergusonii, Klebsiella pneumoniae*) from CD subjects. We have also used the parent consortia CD mucosa-associated microbiota (CD-MAM) from our earlier study^16^ to further characterize how polymicrobial communities are affected by their cultivation in the presence of low concentrations (0.1%) of the two emulsifiers widely used in previous studies: polysorbate 80 and carboxymethylcellulose.

## Results

### Sodium sulfite and polysorbate 80 have profound effects on *F. prausnitzii* growth

**Figure 1.**
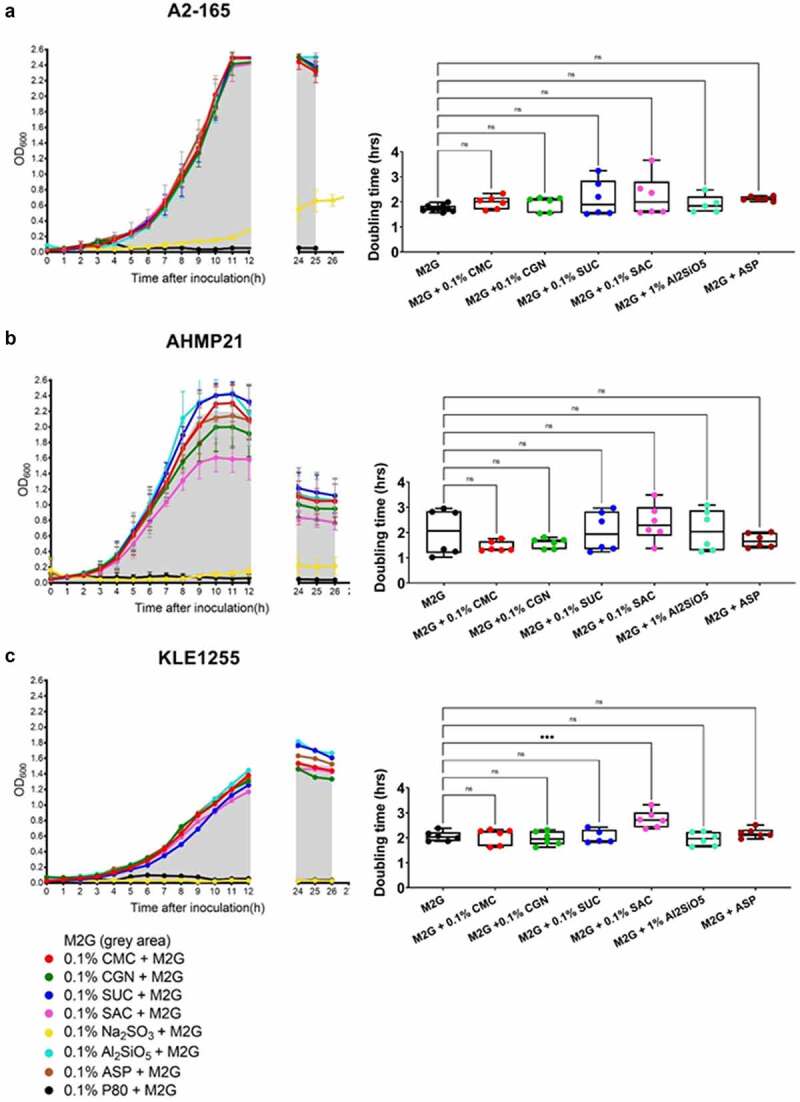
The growth profiles (left) and estimated doubling times (hours, right) of *Faecalibacterium prausnitzii* strains A2-165 (a), AHMP21 (b), and KLE1255 (c) using either the basal medium alone (i.e. M2G broth, denoted by the gray areas) or M2G broth supplemented with 0.1% (w/v or v/v) of different food additives (annotated according to the color-based key). In the panels left, only polysorbate 80 (black) and sodium sulfite (yellow) have profound effects on the growth profiles for all three *F. prausnitzii* strains. In contrast, the other food additives elicit minimal effects on the growth profiles of *F. prausnitzii* strain A2-165 and strain KLE-1255, but appeared to impose some burden on the growth profile of *F. prausnitzii* strain AHMP21. In the panels right, only the doubling time of *F. prausnitzii* strain KLE1255 was significantly increased by the presence of saccharin (*P* ≤ .001). The growth profiles are plots of mean OD_600_ (± SD) values obtained from two rounds of cultivation, each in technical triplicate (n = 6). Doubling times and statistical comparisons were made from the same data, as described in the Methods section.

The growth patterns and calculated doubling times of the three *F. prausnitzii* strains during their culture with either M2G, or with M2G also containing 0.1% concentrations of the different food additives tested, are shown in Figure 1. All three *F. prausnitzii* strains were incapable of growth when glucose was omitted from the basal medium, suggesting none of the food additives alone could support growth (data not shown). For all three strains, both sodium sulfite and polysorbate 80 were found to strongly inhibit growth (Figure 1). Overall, the effects from the other food additives were marginal in terms of affecting the growth profiles and doubling times. Compared to growth on M2G alone, there appeared to be no effect on the doubling times of *F. prausnitzii* strain A2-165 when cultured in the presence of the other food additives tested (Figure 1a, *p* > .05). For *F. prausnitzii* strain AHMP21, the growth profiles suggest that saccharin and perhaps carrageenan impose some metabolic burden on this bacterium, reflected in the reductions in maximal yield compared to the other cultures, although the doubling times were not significantly different to those produced from the control cultures (Figure 1b). Overall, the growth of *F. prausnitzii* strain KLE1255 was slower than the other two strains under all the growth conditions tested, and the addition of saccharin resulted in a small but significant increase in the doubling time for this strain (Figure 1c, *p* < .05). Collectively, these results suggest that sodium sulfite and polysorbate 80 both have a profound inhibitory effect on *F. prausnitzii* growth, and while the other food additives alone cannot support the growth of these strains, the artificial sweetener saccharin may impose a metabolic burden on members of some *F. prausnitzii* phylogroups C (KLE-1255), reflected in the small but significant increases in its doubling times compared to the other *F. prausnitzii* strains examined.

**Figure 2.**
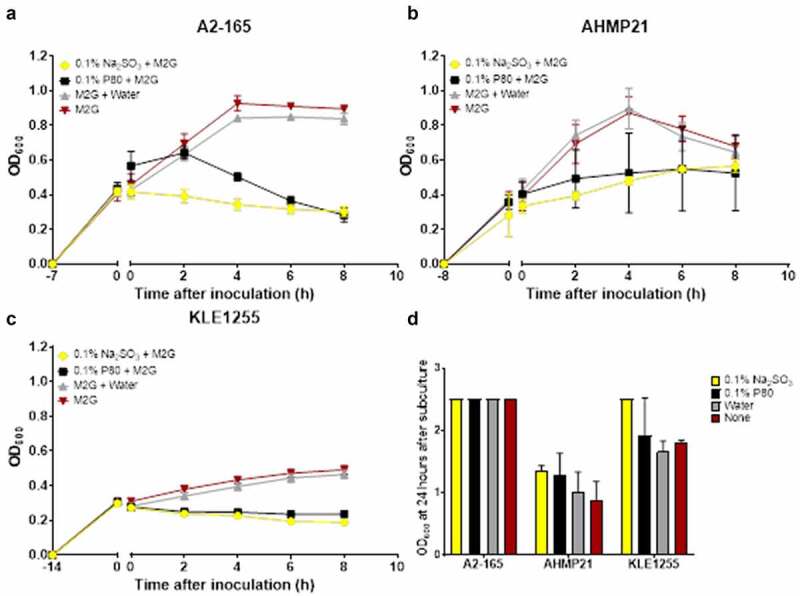
Polysorbate 80 and sodium sulfite appear to exert a bacteriostatic effect on the growth of *Faecalibacterium prausnitzii* strains A2-165 (a), AHMP21 (b) or KLE1255 (c). Here, the OD_600_ of cultures was monitored for 8 hours after either polysorbate 80 (black) or sodium sulfite (yellow) was added to cultures of the *F. prausnitzii* strains during their exponential phase of growth. Cultures receiving either sterile anaerobically prepared water (gray) or no additions (red) were also maintained, to substantiate that any effects on growth were food additive-dependent. Exposure to sodium sulfite strongly arrested the growth of all three *F. prausnitzii* strains for the time course of the study, and while the magnitude of the effect from polysorbate 80 differed among the three strains, in all instances both the growth rate and maximum yield were reduced. Panel D shows the results of separate experiments where subsamples of cultures as described above were used to inoculate tubes of M2G broth medium and the OD_600_ measurements recorded after 24 hours incubation. The growth profiles for the respective strains and cultures represent mean ± SD values obtain from two rounds of cultivation, each in technical triplicate (n = 6).

When either sodium sulfite or polysorbate 80 was added to actively growing cultures of all three *F. prausnitzii* strains growth were arrested for the monitoring period (8 hours) whereas those cultures receiving water alone or no additions showed continued growth over the same period (Figure 2, panels A-C). Aliquots (0.1 ml) of all the respective cultures were then used to inoculate fresh tubes of M2G broth and were incubated for 24 hours. As shown in Figure 2d, all three *F. prausnitzii* strains showed active growth once transferred to broth medium free of sodium sulfite and polysorbate 80, suggesting these food additives exert a bacteriostatic rather than bactericidal effect on *F. prausnitzii* strains.

## Growth of *P. mirabilis* and other *Proteobacteria* are affected by select food additives and oxygen availability

**Figure 3.**
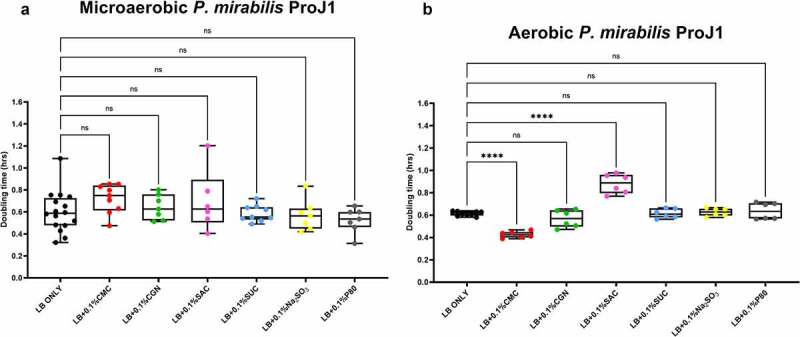
Saccharin and carboxymethylcellulose exert differential effects on *Proteus mirabilis* ProJ1 growth in microaerobic and aerobic conditions. Compared to their growth with the basal medium, microaerobic growth (doubling time, hours) appeared unaffected by any of the food additives tested (panel A). However, its aerobic growth was significantly increased (i.e., reduced doubling time) by the addition of carboxymethylcellulose, but decreased (i.e., increased doubling time) by the addition of saccharin. These data are presented as box plots showing mean ± SD, from no less than two rounds of cultivation, each in technical triplicate (n = 6), with the doubling times calculated and statistical comparisons made as described in the Methods (ns: not significant; ****P ≤ .0001).

**Figure 4.**
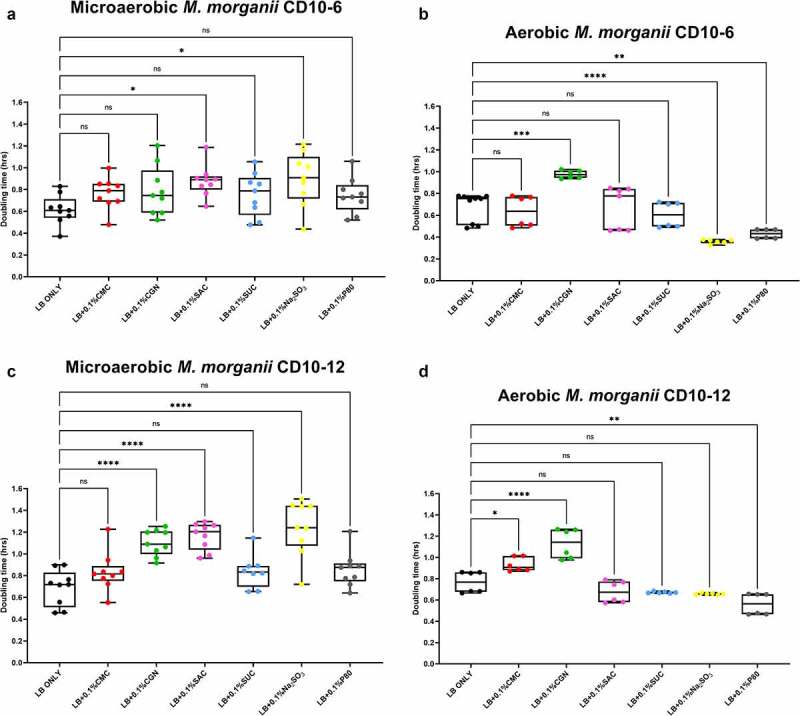
Sodium sulfite, polysorbate 80, saccharin, carrageenan, and carboxymethylcellulose exert differential effects on *Morganella morganii* growth in microaerobic and aerobic conditions. Compared to their growth with basal medium, the microaerobic growth of *M. morganii* strains CD10-6 (panel A) and CD10-12 (panel C) was negatively affected (i.e., increased doubling time) by the addition of either saccharin (*P* ≤ .05) or sodium sulfite (*P* ≤ .001). In contrast, the aerobic growth of both *M. morganii* strains tested was stimulated (i.e., reduced doubling times) by the additions of polysorbate 80 (*P* ≤ .01, panels B and D) and by sodium sulfite for strain CD10-6 (*P* ≤ .0001, panel B). Carrageenan significantly inhibited the aerobic growth of strain CD10-6, and both carrageenan and carboxymethylcelluose significantly inhibited the aerobic growth of strain CD10-12 (panels B and D). These data are presented as box plots showing mean ± SD, from no less than two rounds of cultivation, each in technical triplicate (n = 6), with the doubling times calculated and statistical comparisons made as described in the Methods.

**Figure 5.**
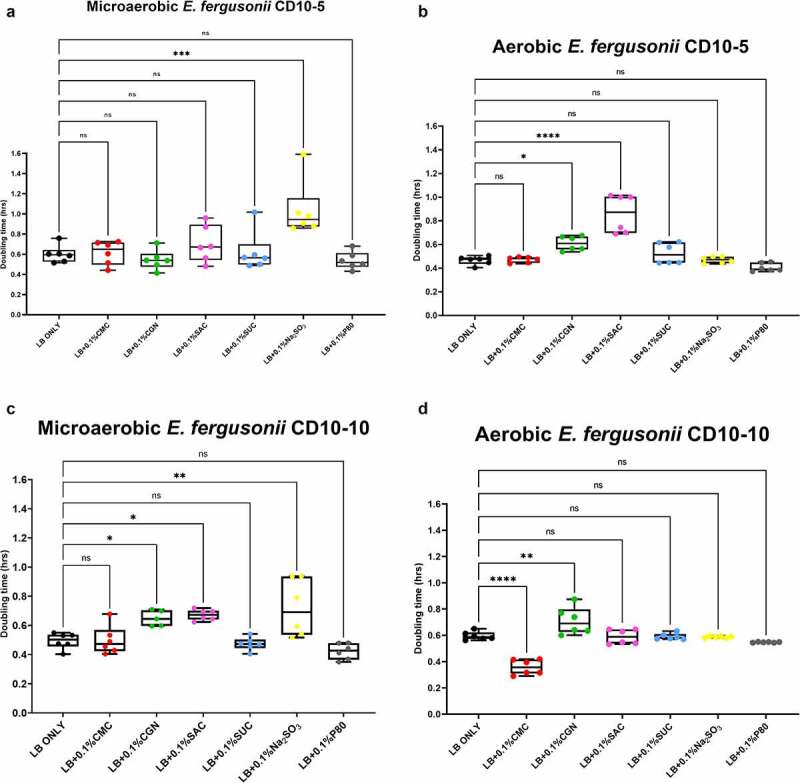
Sodium sulfite, saccharin, and carrageenan exert differential effects on *Escherichia fergusonii* growth in microaerobic and aerobic conditions. Compared to their growth with the basal medium, the microaerobic growth of *E. fergusonii* strains CD10-5 and CD10-10 was negatively affected (i.e., increased doubling time) by the addition of sodium sulfite (*P* ≤ .001, panels A and C) whereas there were no measurable effects on their aerobic growth by this additive (*P* > .05, panels B and D). Carrageenan inhibited the growth (i.e., increased doubling times) of strain CD10-10 under microaerobic conditions (panel C) and for both strains under aerobic conditions (panels B and D). Saccharin tended and significantly inhibited the growth of strain CD10-5 during microaerobic and aerobic growth, respectively (panels A and B) and significantly inhibited the growth of strain CD10-10 under microaerobic conditions (panel C). Interestingly, polysorbate 80 appeared to have no measurable impacts on the microaerobic or aerobic growth of both strains, and carboxymethylcellulose appeared to stimulate the aerobic growth of strain CD10-10 (panel C). These data are presented as box plots showing mean ± SD, from no less than two rounds of cultivation, each in technical triplicate (n = 6), with the doubling times calculated and statistical comparisons made as described in the Methods.

**Figure 6.**
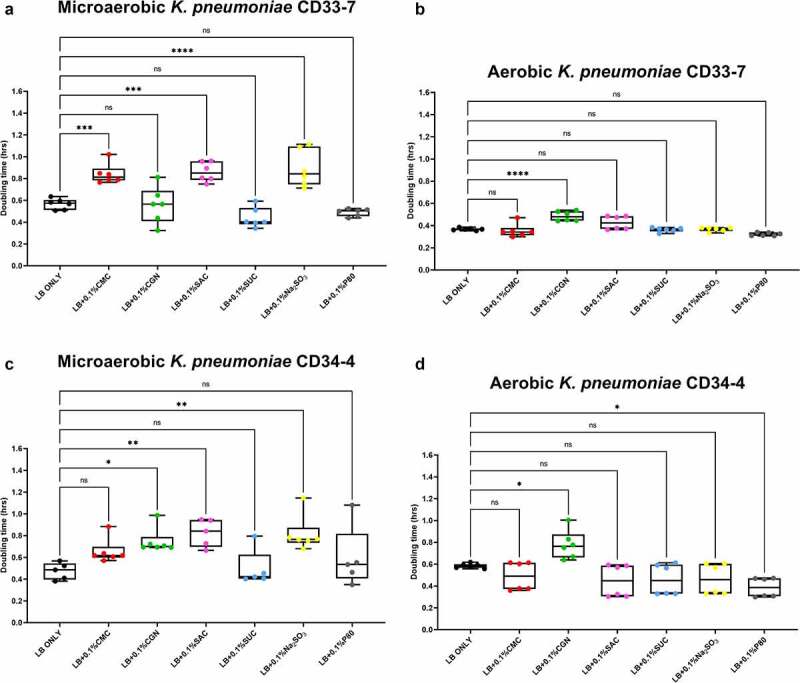
Sodium sulfite, saccharin, and carrageenan exert differential effects on *Klebsiella pneumoniae* growth in microaerobic and aerobic conditions. Compared to their growth with the basal medium, the microaerobic growth of both strains CD33-7 and CD34-4 was inhibited (i.e., increased doubling time) by sodium sulfite and saccharin (panels A and C). Except for microaerobic cultures of strain CD34-4, carrageenan also significantly inhibited the growth of both strains. Polysorbate 80 had limited effects on the growth of both strains except for a stimulatory (i.e., reduced doubling time) effect on the growth of strain CD34-4 in aerobic conditions (panel D). These data are presented as box plots showing mean ± SD, from no less than two rounds of cultivation, each in technical triplicate (n = 6), with the doubling times calculated and statistical comparisons made as described in the Methods.

The results of the growth studies with the *P. mirabilis* and other *Proteobacteria* are shown in Figures 3 through 6. Under aerobic conditions and relative to the LB control cultures, the doubling time of the *P. mirabilis* ProJ1 was significantly altered by the addition of either carboxymethylcellulose (decreased doubling time) or saccharin (increased doubling time). Interestingly though, while the doubling time of the *P. mirabilis* ProJ1 was uniformly reduced under microaerobic conditions, there appeared to be no additional effects on the growth rate of the bacterium (positive or negative) from any of the food additives tested (Figure 3). Despite their close phylogenetic association with *P. mirabilis*, both *M. morganii* strains were affected by a greater range of food additives, which also appeared conditional on oxygen availability (Figure 4). Under aerobic conditions and relative to the LB control cultures, carrageenan, and/or carboxymethylcellulose inhibited the growth of strain CD10-6 and significantly so for strain CD10-12. In contrast, the aerobic growth of both strains was stimulated (i.e., reduced doubling times) by polysorbate 80 and by sodium sulfite for strain CD10-6. However, sodium sulfite had significant and negative effects on the microaerobic growth of both strains; and saccharin also negatively affected the growth of both strains under microaerobic conditions. The effects from the food additives on the growth of both strains of *E. fergusonii* were more diverse (Figure 5). There were no measurable effects from sodium sulfite on the aerobic growth of both strains, but their microaerobic growth was negatively affected (i.e., increased doubling time). In contrast, polysorbate 80 appeared to have no measurable impacts on the aerobic or microaerobic growth of both strains, carboxymethylcellulose appeared to stimulate the aerobic growth (i.e., reduced doubling time) of strain CD10-10, whereas carrageenan inhibited the growth (i.e., increased doubling times) of both strains under aerobic conditions, and the growth of strain CD10-10 under microaerobic conditions. Similarly, saccharin slowed the growth of both strains during aerobic and microaerobic growth, which was significant for strain CD10-5 during aerobic growth, and for strain CD10-10 under microaerobic conditions. The aerobic growth of both strains of *K. pneumoniae* was negatively affected (i.e., increased doubling time) by the addition of carrageenan and while the aerobic growth of strain CD33-7 appeared to be unaffected by the other food additives tested, polysorbate 80 did appear to stimulate (i.e., reduce doubling time) the aerobic growth of strain CD34-4. Under microaerobic conditions, saccharin, and sodium sulfite both negatively affected the growth of both strains; and carrageenan had a negative effect on the growth of strain CD34-4. Sucralose appeared to have no effects on the growth of both strains under aerobic or microaerobic conditions, nor did polysorbate 80 appear to affect the growth of either strain under microaerobic conditions (Figure 6).

In summary, these findings suggest the growth of axenic isolates of Crohn’s disease pathobionts affiliated with the *Morganellaceae* and *Enterobacteriacea* are modulated by commonly used food additives, and further, the impacts of some of these food additives, particularly sodium sulfite, but also carrageenan and saccharin, appear conditional on oxygen availability.

## Polysorbate 80 and/or carboxymethylcellulose have a “personalized” effect on the Crohn’s disease mucosa-associated microbiota

**Figure 7.**
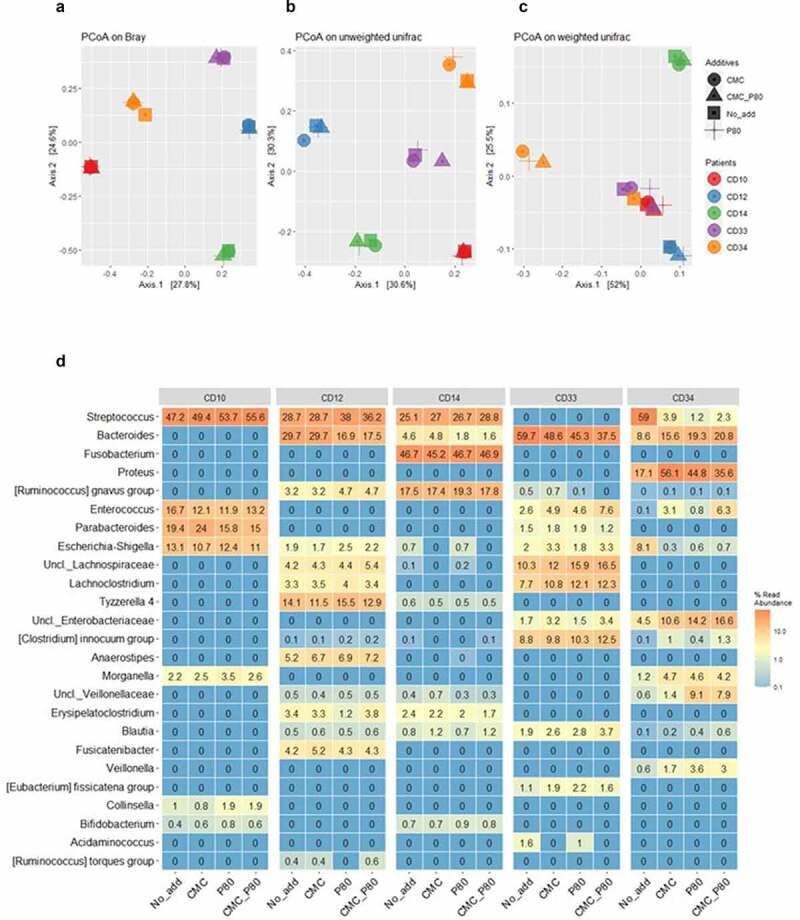
Principal Coordinates Analysis (PCoA) of the Bray Curtis dissimilarity (a), unweighted (b) and weighted (c) UniFrac metrics of the mucosa-associated microbiota from 5 post-operative CD patients, following their culture in habitat-simulating medium containing either no additions, or 0.1% final concentrations of either P80 and/or CMC. The legend for subject and medium composition are shown in panel A. The results suggest only the weighted UniFrac metric of the microbial consortia from subject CD34 undergoes dramatic changes in response to P80 and/or CMC. Panel D shows the heatmap displaying the top 25 predominant bacterial taxa (expressed as % read abundance) present in the microbial communities recovered from the 5 post-operative CD patients following growth with either basal medium (No_add), or the basal medium supplemented with 0.1% (final concentration) of either carboxymethylcellulose (CMC), polysorbate P80 (P80), or both food additives (CMC_P80). Note the subject consortium-specific effects from the food additives on members of *Streptococcus* and *Bacteroides* spp., and particularly for CD34, the effects from P80 on the increased relative abundances of *Veillonella*, unclassified Family*_Veillonellaceae* (Uncl.*_ Veillonellaceae*), and *Morganella* spp. in cultures emended with P80 only. There is also an increased relative abundance of *Proteus*, unclassified Family_*Enterobacteriaceae* (Uncl.*_ Enterobacteriaceae*), and *Enterococcus* spp. within cultures containing P80 and/or CMC, and reductions in the relative *Escherichia-Shigella* in response to these additives. Panel d reproduced with permission from Kang et al.^55^

We next sought to establish the effects from select food additives on more complex communities of the mucosa-associated microbiota (MAM) recovered from CD patients. To that end, the CD-MAM from five different CD subjects were challenged with either no additions, or 0.1% (final concentrations) of polysorbate 80 and/or carboxymethylcellulose, and the profiles of the communities following their cultivation are shown in Figure 7. The unsupervised analyses of beta diversity metrics for the respective cultures showed the patient specificity of the CD-MAM is essentially retained and there are limited intra-subject variations in response to the food additives tested as measured via Principle Coordinate Analysis (PCoA) of the Bray-Curtis dissimilarity (panel A, Axes 1 and 2 account for 52.4% of the total variation) or unweighted UniFrac (panel B, Axes 1 and 2 account for 60.9% of the total variation). However, there are some remarkable changes revealed via PCoA analysis of the weighted UniFrac metrics (panel C, Axes 1 and 2 account for 77.5% of the variation). Here, the CD-MAM consortia recovered from subjects CD10, CD33, and CD34 are clustered together, and while some modest alterations are apparent in response to either or both food additives for the consortia from CD10 and CD33, there are profound changes in the CD-MAM consortia from patient CD34 in response to exposure to polysorbate 80 and/or carboxymethylcellulose.

The relative abundance data (genus level) for the CD-MAM consortia following their culture in basal M2GSC medium (control), or in the presence of polysorbate 80 and/or carboxymethylcellulose, are shown in Figure 7d. These data are consistent with the unsupervised (PCoA) analyses of the beta diversity metrics but provide greater insights into the subject/bacterium specific alterations in the CD-MAM. Specifically, the changes in the PCoA plots of the weighted UniFrac metrics seen for the CD-MAM from subject CD34 in response to polysorbate 80 and/or carboxymethylcellulose is explained by the profound increase in the relative abundance of the genera *Proteus* and *Morganella*, unclassified Family_*Enterobacteriaceae*, and key members of the *Negativicutes*: unclassified Family_*Veillonellaceae* and *Veillonella* spp. Concomitantly, there were substantial reductions in the relative abundances of *Streptococcus* spp., and *Escherichia-Shigella* spp. in the CD34 consortium. Polysorbate 80 alone also resulted in a reduction (CD12, CD14) or expansion (CD34) in the relative abundances of members of the genus *Bacteroides*, which are linked with a concomitant expansion (CD12) or reduction (CD34) in members of the genus *Streptococcus*. Similarly, carboxymethylcellulose *per se* is associated with an expansion of the relative abundance of *Parabacteroides* spp. (in CD10 but not CD33), *Enterococcus* spp. (CD33 and CD34, but not CD10) and unclassified Family_*Enterobacteriaceae* (CD33 and CD34).

Collectively, these findings suggest that in 4 of 5 CD subjects, the impacts from polysorbate 80 and/or carboxymethylcellulose were relatively modest, and the microbial consortia retained their patient specificity, rather than a convergence of the microbial communities toward a common collection of bacterial taxa in response to either or both food additives. However, and exceptionally, subjects possessing specific bacterial taxa can show profound changes in the composition of their mucosa-associated microbiota. In this specific example, it appears that the MAM communities from subjects with detectable *Veillonellaceae, Veillonella* spp., *Morganella* spp., and *Proteus* spp. (CD34) can show a profound increase in the relative abundances of these bacterial taxa, in response to relatively small concentrations of polysorbate 80 and/or carboxymethylcellulose.

## Discussion

Microbial dysbiosis is widely considered a hallmark of IBD, typified by a depletion of the relative abundance of bacteria affiliated with the *Lachnospiraceae* and *Ruminococcaceae* and a commensurate expansion in the relative abundance of bacteria affiliated with the *Proteobacteria*.^32–34^ Furthermore, a limited number of longitudinal studies have also shown restoration of *F. prausnitzii* in the colonic microbiota is associated with promoting disease remission.^11^ Indeed, depleted *Faecalibacterium* spp. combined with detectable *P. mirabilis* populations are now strongly associated with disease recurrence^18,^^19^ and the *Negativicutes* (e.g., *Veillonellaceae*) as well as Fusobacteria are enriched in CD patients that show early post-operative endoscopic recurrence.^23^ However, the ecological dynamics giving rise to these observations remain largely unexplained, and thereby difficult to predict or manage. Diet has emerged as key factor affecting the nutritional and physiological ecology of the gastrointestinal tract and can function both as trigger and as a treatment option for chronic and idiopathic conditions such as CD.^24^ In that context, along with the plethora of processed foods now available to consumers, the diversity and consumption of food additives that enhance taste, flavor, and sensory perceptions have also increased. Evidence linking the consumption of food additives with host- and/or microbial alterations characteristic of disruptions to gut homeostasis and inflammation is mounting, but the actual mechanisms driving these observations are still lacking.

The food additives tested in this study represent four different classes (preservatives, sweeteners, thickeners, and emulsifiers) and chemistries (organic, inorganic, small molecules, and polymers). Our choice of challenging the *F. prausnitzii* and *Proteobacteria* strains with 0.1% final concentrations of these additives is considerably less than that used in some *in vivo* animal studies and *in vitro* studies.^30,35^ However, our choice is based on the strict regulatory limits on the amounts of specific food additives that can be included in human foods, their recommended average daily intakes where available^36,37^ and our estimation of their dilution within the luminal contents of the colonic environment. At this concentration, only sodium sulfite and polysorbate 80 elicited profound effects on the *F. prausnitzii* strains examined here, while the other food additives had variable (strain-specific) effects on the *Proteobacteria* strains tested, which were also affected by oxygen availability during growth. Interestingly, while both polysorbate 80 and sodium sulfite were inhibitory to nascent and actively growing cultures of multiple *F. prausnitzii* strains, polysorbate 80 appeared to have either little or a growth-promoting effect on the axenic isolates of *Proteobacteria*, and the effects from sodium sulfite on the growth of these strains were conditional oxygen availability. Sulfites have long been used as an antimicrobial agent to prevent browning and food spoilage^38^ but there are relatively few studies examining the potential antimicrobial effects of this additive on the “commensal” gut microbiota.^39^ While an acceptable daily intake (ADI: 10ng/kg) of sulfites has been in place for nearly 50 years, Irwin *et al*.^38^ suggested that an “average” Western diet can result in double the recommended ADI of sulfites; and will elicit bactericidal or bacteriostatic effects on four of the commonly used probiotic strains, at concentrations regarded as safe by the FDA. Interestingly, the results with the *Proteobacteriaceae* strains collectively show that the inhibitory effect of sodium sulfite is conditional on their growth under “microaerobic” conditions. Taken together, the consumption of foods/diets resulting in sulfite concentrations ~0.1% (w/v) in the gut milieu would at least transiently suppress *F. prausnitzii* growth within the colonic microbiota and reduce the growth rates of at least some strains of *Proteobacteria*, provided oxygen availability is limited, as it should be during periods of gut homeostasis.

Interestingly, while recent studies with healthy subjects suggest inclusion of carboxymethylcellulose to an additive-free diet leads to reductions in the relative abundance of *F. prausnitzii*^40^ our findings suggest the impacts from this additive are not direct, but rather indirect via niche contraction and/or occupation. The artificial sweetener saccharin did result in a metabolic burden placed on the growth of *F. prausnitzii* strain AHMP21, and a small but statistically significant reduction in the growth of *F. prausnitzii* strain KLE-1255, whereas sucralose had minimal effects on all three strains, relative to the control cultures (Figure 1). When administered at greater concentrations than those used here, a chronic daily intake of sucralose (as Splenda) was found to reduce total anaerobic bacterial counts in rats^41^ and oral ingestion of saccharin by mice also leads to an under representation *Clostridium-*affiliated lineages in animal models.^42^ However, an assessment of the available *in vivo* studies examining the health effects of artificial sweeteners concludes that at the current ADI recommendations, artificial sweeteners are unlikely to drive substantive alterations to the gut microbiota.^43^ Our findings with both *F. prausnitzii* and the *Morganellaceae* and *Enterobacteriacea* lineages are not inconsistent with such conclusions but suggest even relatively small concentrations of sweeteners may elicit subtle effects on the gut microbiota, perhaps via adding a metabolic burden that negatively affects the growth efficiency of at least some strains of bacteria. Dietary patterns that favor a relatively high intake of artificial sweeteners may affect the ecological resilience and restoration of select bacteria in patients with CD.

Since the findings by Chassaing *et al*^44^ showing the consumption of polysorbate 80 (1% v/v in drinking water) by mice promotes structural and microbiological changes leading to gut inflammation, this food additive has received greater investigation about its potential health impacts. For example, Singh *et al*.^45^ have also found that polysorbate 80 administered by gavage not only induced gut inflammation and liver dysfunction but also reduced fecal short-chain fatty acid concentrations in mice. Gerasimidis *et al*.^30^ and Naimi *et al*.^46^ both reported that polysorbate 80 added to mixed bacterial cultures produced from fecal samples of healthy adults reduces the relative abundance of *F. prausnitzii*. Here, our studies using multiple axenic isolates *F. prausnitzii* show the effect of 0.1% (v/v) polysorbate 80 (and sodium sulfite) is direct and likely bacteriostatic to this bacterium, at a concentration representing a “real-world” intake of this food additive. Conversely, polysorbate 80 appears to have little or no inhibitory effects toward the urease producing *Proteobacteria* examined here and indeed, appears stimulatory to the growth rate of axenic cultures of *M. morganii* and *K. pneumoniae.*

The composition of the mucosa-associated microbiota is not only different to that present within the fecal stream and/or stool in CD subjects^47^ but also not subject to the growth constraints imposed from digesta flow. As such, the use of fed batch cultures to examine these communities provides a realistic approach to examine the dynamics of these communities. Our results with the consortia recovered from five different CD patients show that the most profound effects from polysorbate 80 and/or carboxymethylcellulose was “personalized” toward the communities possessing *P. mirabilis, M. morganii* and/or *Veillonella* spp. In that context, polysorbates are well recognized to support the growth of *Veillonella* spp.^48^ which are a numerically predominant member of the *Negativicutes* lineage of gut bacteria. It is clinically salient to note that the relative abundance of *Veillonella* spp. in 16S rRNA gene profiles of mucosal tissue and stool metagenomic sequence data were positively associated with new onset, treatment-naive CD and more severe lesions^47^ and also in CD patients with early post-operative endoscopic recurrence of disease.^23^ With respect to *P. mirabilis*, in addition to our own studies and others showing the links between this bacterium and CD pathogenesis^21^ as well as disease recurrence,^18,^^19^ there is emerging evidence that expansion of *Proteus* spp. within the gastrointestinal microbiome is linked with heightened inflammation and/or tumorigenesis. For instance, recent studies showed that the addition of 1% (v/v) polysorbate 80 in drinking water resulted in an expansion of the relative abundance of *“Enterobacteriaceae”* in ileal samples of mice and potentiated their susceptibility to indole-methicin induced damage to the epithelium.^49^ Culture of ileal contents revealed that *P. mirabilis* counts were specifically and significantly increased in those mice consuming polysorbate 80. There is also an expansion of the relative abundance of *Proteus* spp. in association with the provision of a “Western diet” to a mouse model of tumor formation following colorectal surgery, which was potentiated by the emendment of the mouse gut microbiome with *Enterococcus* spp.^50^ The authors note the collagenolytic activity of these two genera as a key metabolic feature contributing to tumor formation, so it is interesting to also note that the relative abundances *Enterococcus* spp. were also found to increase in those patient-specific mucosa-associated consortia containing these lineages.

In conclusion, we have examined the direct effect from four different classes of food additives commonly used in western foods on the growth of bacteria linked with Crohn’s disease remission or recurrence. The collective results with axenic isolates suggest that, with the exceptions of sodium sulfite and polysorbate 80, the effects from the other food additives examined here on bacterial growth rates appear to be strain-specific rather than universal. Our findings most clearly demonstrate that sulfites and polysorbate 80 at relatively small concentrations elicit a direct and profound bacteriostatic effect on multiple strains of *F. prausnitzii* and thereby, its contributions to the prevention of, and recovery from, inflammation. Interestingly, the magnitude of the effects from sodium sulfite and polysorbate 80 on the *Proteobacteria* isolates examined here appear to be contingent on oxygen availability. By using mucosa-associated microbial consortia recovered from the biopsies of five CD subjects, we have not only validated key findings using axenic isolates, but also show that two different emulsifiers (polysorbate 80 and carboxymethylcellulose) – either alone or in combination – can produce a “personalized” impact on the composition of these communities, including bacterial taxa such as *Proteus* spp. and *Negativicutes*, which are implicated in promoting CD recurrence. This study reveals how some of the key bacterial taxa now being identified as biomarkers of CD recurrence, or remission, will respond to different food additives, which is an important step toward diet formulation in CD therapy and clinical management.^51^

## Materials and methods

### Bacterial strains and consortia and standard growth conditions

*Faecalibacterium prausnitzii* strain A2-165 (Accession: SAMN07362387), KLE-1255 (Accession: SAMN00189147) and AHMP21 (Accession SAMN07414879) representing Phylogroups B, C, and E, respectively^52^ were routinely cultured with either M2GSC agar as described by Miyazaki *et al*^53^ or a basal broth (M2) medium with only glucose added, designated M2G. All bacterial transfers and growth were performed under anaerobic conditions, either within an anaerobic chamber on agar plates (Coylabs, Detroit, MI, atmosphere of 75N_2_:20CO_2_:5H_2_) or as broth cultures within Hungate tubes, with the same gas mixture used in the headspace.

The production of the microbial consortia from tissue biopsies and the subsequent isolation of urease positive *Proteobacteria* from these consortia have been described in detail by Teh et al.^16^ Briefly, tissue was collected using standard forceps from 5 CD subjects during their post-operative endoscopy as part of the Post-Operative Crohn’s Disease Endoscopic Recurrence (POCER) study.^54^ The tissue was transferred to sterile and anaerobically prepared cryopreservative and the microbial consortia propagated from individual tissue samples by 24 h culture with M2GSC broth medium as previously described^16,^^54^ and stored as glycerol-preserved stocks. Urease-positive clinical isolates were recovered from serial dilutions of these glycerol stocks as individual colonies developing on either MacConkey or Cysteine-, Lactose-, Electrolyte deficient agar plates (Oxoid Ltd, UK) under aerobic conditions. Urease-positivity was confirmed via color change surrounding colonies on Urea Agar Base plates with added Phenol Red and 33 isolated colonies were re-plated to produce axenic cultures. The taxonomic assignment of the axenic isolates was confirmed by 16S rRNA gene amplification and Sanger sequencing.^16^ Following preliminary testing, an isolate of *P. mirabilis*, and 2 strains each taxonomically classified as *Morganella morganii, Escherichia fergusonii*, and *Klebsiella pneumoniae* were selected for further study.

### Preparation of food additive stock solutions

The food additives tested in this study were purchased from Sigma-Aldrich (Castle Hill, Australia). Solutions of carboxymethylcellulose sodium salt (21904), ι-carrageenan (C1138), saccharin (109185, ≥ 99%), sucralose (69293, ≥98.0%), sodium sulfite (S0505, ≥98%), Tween (polysorbate) 80 (polysorbate 8074), aluminum silicate (520179), and aspartame (A5139) were prepared at 1% (w/v or v/v) concentration in milli-Q water and flushed with N_2_ for an hour. The solutions were transferred to an anaerobic chamber (N_2_:H_2_, 95:5) and transferred to glass serum bottles sealed with a rubber stopper and crimp sealed. These solutions were then autoclaved and stored at room temperature prior to use.

For the growth studies with the *F. prausnitzii* strains, the food additive solutions were used to supplement the basal M2G medium by aseptically transferring 1 ml of each stock solution to individual tubes, to provide final concentrations of 0.1% (w/v or v/v), which is intended to approximate concentrations found within the gut milieu. The same approach was used with the *Proteobacteria* strains with the exception that Luria Bertani (LB) broth was used as the basal medium.

### Bacterial growth assays

The growth experiments were repeated at least twice and with three technical replicates for each treatment group. For initial growth experiments, a single colony of each *F. prausnitzii* strains was transferred to M2G broth and incubated at 37°C overnight (14 − 16 hours). Aliquots (100 μl) of these cultures were used to inoculate Hungate tubes containing anaerobically prepared M2G broth medium supplemented with either the food additives, or additional sterile water, to provide a final volume of 10 ml. Microbial growth was assessed hourly by measuring the culture optical density at 600 nm (OD_600_). Based on the results, a second series of experiments with all three *F. prausnitzii* strains were performed. All three strains were cultured with M2G broth and growth monitored as described above. Once the cultures had reached mid-exponential phase of growth, either the sodium sulfite or polysorbate 80 stock solutions were added to provide a final concentration of 0.1% and bacterial growth monitored longitudinally for another 8 hours, as described above. As controls, separate cultures received a similar volume of sterile anaerobic water or were left untreated.

The growth studies with *Proteobacteria* strains were conducted within a 96-well microtiter plate format. Here, the inoculating cultures were prepared by using a single colony of each strain transferred to 5 ml of LB broth within a 50 ml centrifuge tube (Corning, US) and incubated for 5 hours aerobically with shaking at 200 rpm at 37°C. Aliquots (0.135 ml) of either sterile LB broth, or LB broth prepared to contain 0.1% final concentration one of the food additives described above (with the exceptions of aluminum silicate and aspartame) were dispensed into individual wells, which were then inoculated with 0.015 ml of a 1:100 dilution (with LB) of the inoculating cultures described above. As controls, wells containing 0.15 ml of uninoculated broth (with and without food additives) were also prepared and positioned around the edge of the microtiter plates. The plates were covered with Breathe-Easy sealing membranes and placed within a Multiskan GO microplate reader (ThermoFisher, US) for measurements of aerobic growth. The plate reader was programmed to shake at 300 rpm and OD_600_ measurements were taken every 30 minutes for 17 hours. A parallel series of growth studies were also done with the 7 *Proteobacteria* strains using a 96-well microtiter plate format under “microaerobic” conditions. Here, the LB medium was prepared with an additional step of gassing the solution with a steady stream of nitrogen gas for 60 minutes, and the manipulations described above were performed within a Coy Anaerobic chamber filled with a mixture of CO_2_:H_2_:N_2_ (15:5:80). The “microaerobic” conditions resulted from the periodic introduction of surrounding atmosphere during interchange operations, which resulted in transient readings of ~300 ppm oxygen within the chamber atmosphere, before returning to zero. Bacterial growth was monitored by OD_600_ measurements using a FLUOStar Omega (BMG Labtech, Germany) plate reader permanently housed within the chamber using the same operational parameters described above.

### Studies with CD patient-specific microbial consortia

Overnight cultures of each consortium were used to inoculate the same M2GSC medium prepared to contain either 0.1% (w/v) carboxymethylcellulose, 0.1% (v/v) polysorbate 80, or both. Control cultures (i.e., no additives) were also prepared. After incubation for 24 hours, the resulting cultures were sampled, pooled, and metagenomic DNA extracted and initially used for 16S rRNA gene amplicon sequencing.^16^

### Statistical and bioinformatic analyses

For studies with axenic bacterial isolates, the doubling times were calculated using the Malthusian growth model, applied to OD_600_ measurements representing the exponential phase of growth from individual cultures (technical triplicates) and no less than two rounds of growth studies. Multiple comparisons were performed using ANOVA with Dunnett’s comparison test in GraphPad Prism 9.1.0. Statistical analyses with *p*-values less than 0.05 were considered significant. For the consortia-based studies, the production of 16S rRNA gene amplicon libraries, DNA sequencing and data filtering were processed using the QIIME2 software, and the taxonomic profiles and comparisons were computed using various R packages, all as described by Teh et al.^16^ The DNA sequencing data (fastq files) produced as part of this study are uploaded in NCBI SRA as accession number PRJNA779743.

## Data accession

The DNA sequencing data (fastq files) produced as part of this study are uploaded in NCBI SRA under accession number PRJNA779743.
